# Very low prevalence of *Plasmodium falciparum* histidine-rich protein 2 (*pfhrp2*) gene deletion in the Brazil, Venezuela, and Guyana tri-border

**DOI:** 10.1038/s41598-024-83727-3

**Published:** 2025-01-03

**Authors:** Maria Eduarda Pereira Mascarenhas, Jaime Louzada, Renato Amorim Rosa, Gabriela Maíra Pereira de Assis, Flora Satiko Kano, Joseli Oliveira-Ferreira, Tais Nobrega de Sousa

**Affiliations:** 1 Molecular Biology and Malaria Immunology Research Group, Instituto René Rachou (IRR), Fundação Oswaldo Cruz (FIOCRUZ), Minas Gerais, Brazil; 2 Federal University of Roraima, Roraima, Brazil; 3 Immunoparasitology Laboratory, Instituto Oswaldo Cruz (IOC), Fundação Oswaldo Cruz (FIOCRUZ), Rio de Janeiro, Brazil; 4 Department of Microbiology, Tumor and Cell Biology, Karolinska Institutet, Solna, Sweden

**Keywords:** Malaria, *Plasmodium falciparum*, *pfhrp2*, *pfhrp3*, RDT, Genetic diversity, Parasite genetics, Diagnostic markers, Malaria

## Abstract

Rapid Diagnostic Tests (RDTs) have been an important diagnostic tool for detecting *P. falciparum* malaria in resource-limited settings. Most tests are designed to detect the Histidine-rich Protein 2 (HRP2). Parasites lacking *pfhrp2* and its homologous *pfhrp3* have been reported in several regions, with prevalence reaching 100% in certain areas. To better characterize *P. falciparum* isolates circulating in the Brazil-Venezuela-Guyana tri-border region, we performed a comprehensive analysis of 365 samples collected between 2016 and 2018. Molecular and immunological methods were employed to detect HRP2 and confirm *pfhrp2/3* deletions. Our findings point to a low prevalence (1%) of *pfhrp2*-deleted parasites confirmed by the lack of HRP2 detection. Among false-negative HRP2-RDT tests (6%), most were attributed to low parasite densities. A merozoite surface protein 2 (*msp2*)-based intra-host diversity analysis suggested overall low genetic diversity. The pattern of HRP2 sequences resembled that has been previously described in areas along the Brazil and French Guiana border. In conclusion, we have found a low prevalence of *pfhrp2*-deleted parasites in the north-central Guiana Shield, which contrasts with the findings reported at the Peru border. Continued surveys are necessary to monitor the prevalence of *pfhrp2* deletion in this area characterized by a high number of cross-border malaria cases.

**Supplementary Information:**

The online version contains supplementary material available at 10.1038/s41598-024-83727-3.

## Introduction

In 2022, approximately 131,000 cases of malaria were recorded in Brazil, with 84% caused by *P. vivax* and 14% by *P. falciparum*^1^. In the first half of 2023, there was an 8.7% increase in malaria cases compared with the same period in the previous year, especially in indigenous (34.8%) and mining areas (11.2%). Brazilian gold miners spend part of their time between mines abroad and in Boa Vista, Roraima State, in northern Brazil, generating high mobility between malaria-endemic areas in Venezuela and Guyana^2^. Imported malaria is considered a critical obstacle to achieving elimination in many countries, including Brazil^2^.

An accurate and timely diagnosis is fundamental for adequate treatment of patients and the prevention of mortality. For this reason, since 2010, the World Health Organization (WHO) has recommended that all individuals suspected of having malaria should undergo parasitological confirmation through microscopy or Rapid Diagnostic Tests (RDTs) before initiating treatment^3^. Since then, RDTs have become a crucial tool, especially in places where there is a lack of experienced microscopists and adequate infrastructure^3^. Histidine-rich Protein 2 (HRP2) is the main target of the 415 million *P. falciparum* detection RDTs sold annually^4^. Due to its high expression levels, HRP2-based detection is more sensitive than the detection of another commonly used antigen, such as pan-*Plasmodium* lactate dehydrogenase (pLDH)^5^.

Parasites that do not express HRP2 cannot be detected by the RDTs based on this antigen. Nonetheless, HRP2-based RDTs can cross-react with the Histidine-rich Protein 3 (HRP3), masking the effects of *pfhrp2* deletion^6^. In recent years, parasites with deleted *pfhrp2/3* have been documented on different continents, with prevalence estimates varying widely within and between countries^7^. In South America, the highest reported rates were in Peru and the border region of Brazil and Peru^8,9^, with the prevalence of dual *pfhrp2/3*-deleted parasites ranging from 46 to 79.8%. Lower prevalences of *pfhrp2* deletion were reported in the eastern Brazilian Amazon, such as in Roraima State (15%), with no *pfhrp2-*deleted parasites in the far east Amapá e Pará States^10,11^. In this study, we aimed to assess the prevalence of *pfhrp2/3* deletion in order to gain a better understanding of the genetic profile of *P. falciparum* isolates circulating in the border areas among Brazil, Venezuela, and Guyana, in the north-central Guiana Shield. Our findings indicate that the prevalence of *pfhrp2* deletion is very low in this area, which was characterized by intense migration of Venezuelans during the study period. Most of the false-negative HRP2-based RDT results were due to low-density infections.

## Results

### Positivity rates of HRP2 in the RDT

**Fig. 1:**
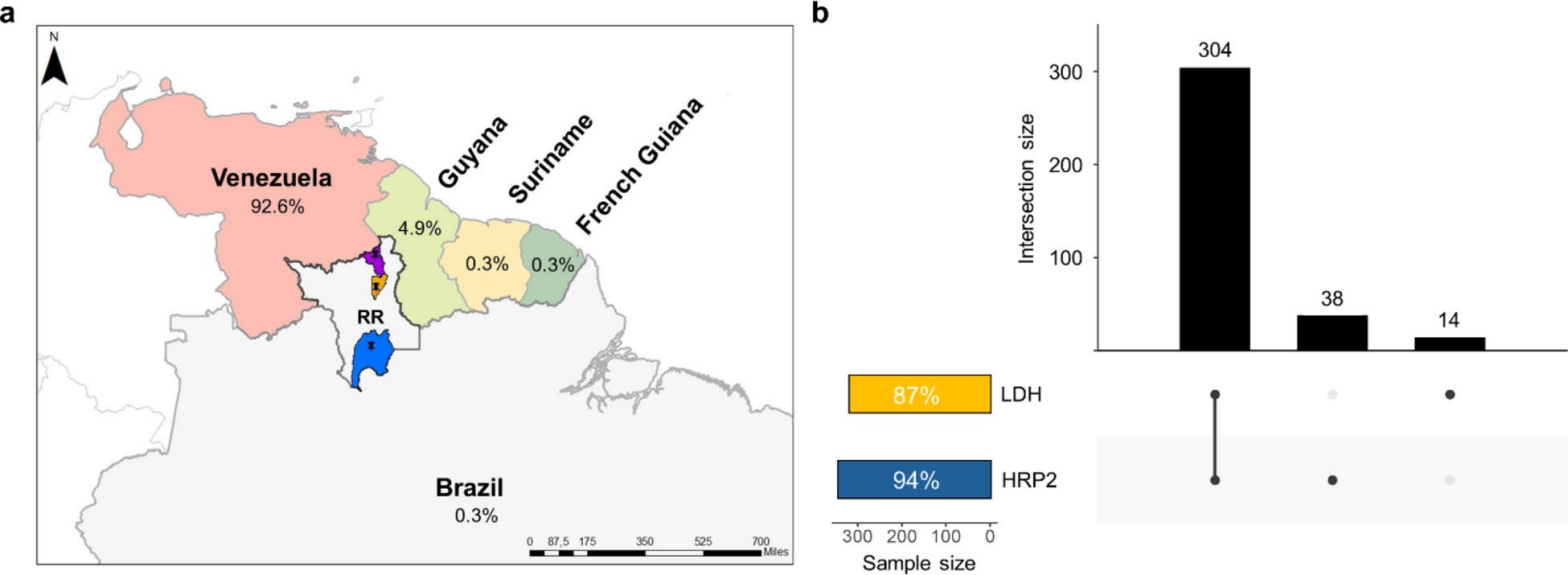
Sites of sample collection and infection, and the rate of positivity in rapid diagnostic tests (RDT). **(a)** A map of the north region of Brazil shows the probable infection sites of participants enrolled in this study. The sample collection sites in Brazil were Rorainópolis (indicated in blue), Boa Vista (indicated in orange), and Pacaraima (indicated in purple) cities in Roraima (RR) State. The site of infection was unknown for 1.6%. The map was made with ArcGIS software. **(b)** Rate of positivity in RDT for HRP2 (in blue) and LDH (in yellow).

We evaluated a total of 365 samples that were positive for *P. falciparum* by optical microscopy and later confirmed by PCR. Most participants were adult males (70%) infected in mining areas in Venezuela (92.6%) and Guyana (4.9%) (Fig. 1a). Of the total number of samples, 342 (94%) were positive in the RDT for HRP2 and 318 (87%) for LDH (Fig. 1b). Three hundred and four (83%) samples were positive for both antigens, 38 (10%) were positive only for HRP2 and 14 (4%) were positive only for LDH (Fig. 1b). Of note, 9 (2.5%) samples were undetectable by the RDT (negative for both HRP2 and LDH), which presented a median parasitemia of 180 parasites/µL (IQR = 120–1480). The lowest reported parasite density was 10 parasites/µL, as estimated by microscopy.

**Fig. 2:**
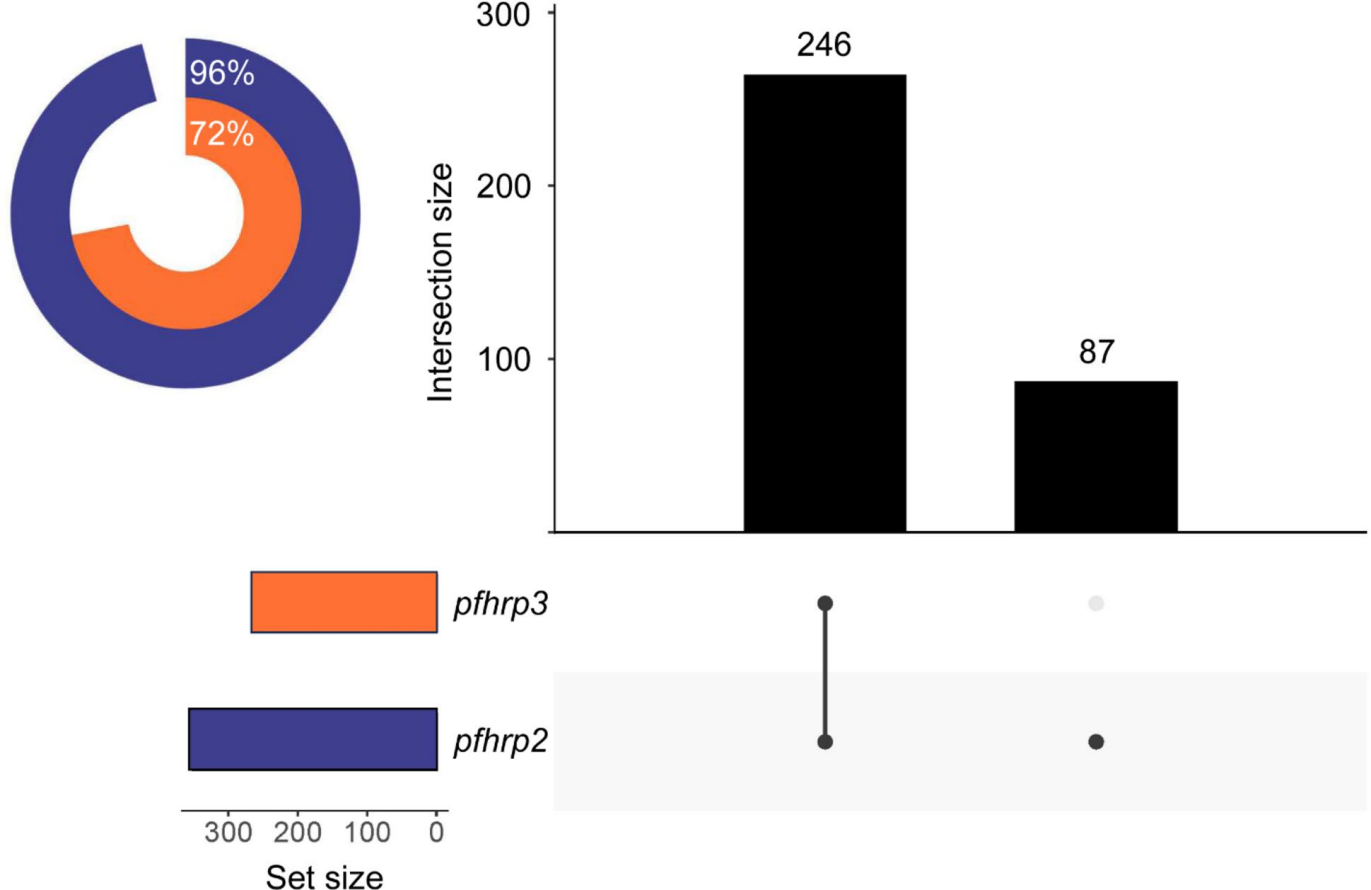
Positivity rates for the *pfhrp2* and *pfhrp3* assayed by nested PCR. The circular graph shows the relative frequency of positive samples for each of the genes. The absolute numbers of *pfhrp2/3-*positive samples are presented in the bar plot. The *pfhrp2* samples are shown in blue and the *pfhrp3* samples are in orange.

### nPCR detection of *pfhrp2* and *pfhrp3*

We observed a significant difference in the deletion rates between *pfhrp2* and *pfhrp3* (Fig. 2). For the *pfhrp2* gene, 351 (96%) of the samples were amplified, while for *pfhrp3*, only 264 (72%) showed a positive result on the agarose gel. In all 14 cases where the *pfhrp2* gene was not amplified, the *pfhrp3* gene was also absent. All *pfhrp2-* and *pfhrp3-*negative samples were subjected to further analysis to determine the presence of the flanking genes located upstream and downstream of the two target genes (Supplementary Data 2). In the case of *pfhrp2*, the MAL7P1.230 flanker was absent in 7 out of 14 (50%) samples, as well as the MAL7P1.228 flanker. For *pfhrp3*, the MAL13P1.475 flanker was absent in 56 out of 101 (55%) *pfhrp3-*negative samples, while the MAL13P1.485 flanker was the most absent gene, with only 6 (6%) positive samples for this target.

### Comparison between molecular methods for the detection of *pfhrp2*

**Fig. 3:**
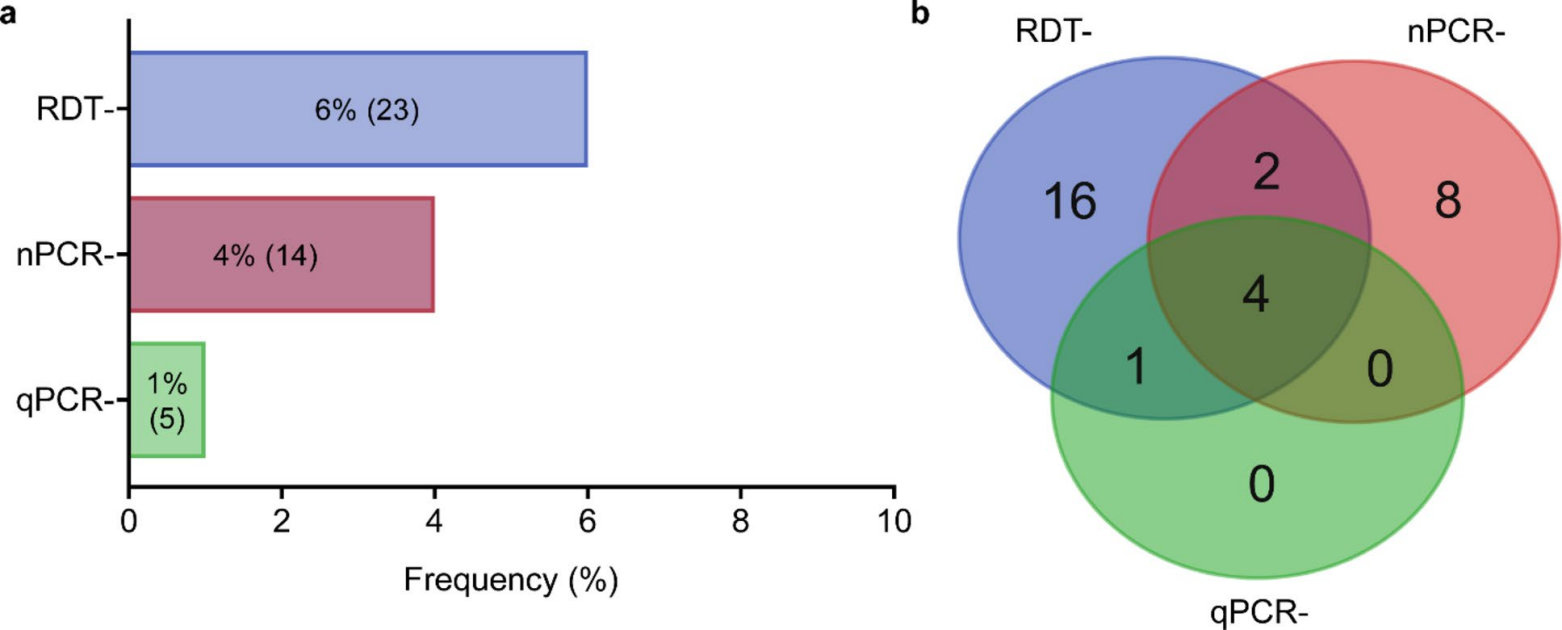
Frequency of negative tests for the detection of *pfhrp2* (nPCR and qPCR) or HRP2 (RDT) for samples analyzed from 365 subjects with symptomatic malaria by *P. falciparum*. **(a)** Bar graph showing the negative rates. Absolute frequencies are in brackets. **(b)** A Venn diagram illustrates the degree of concordance between molecular tests and RDTs.

**Fig. 4:**
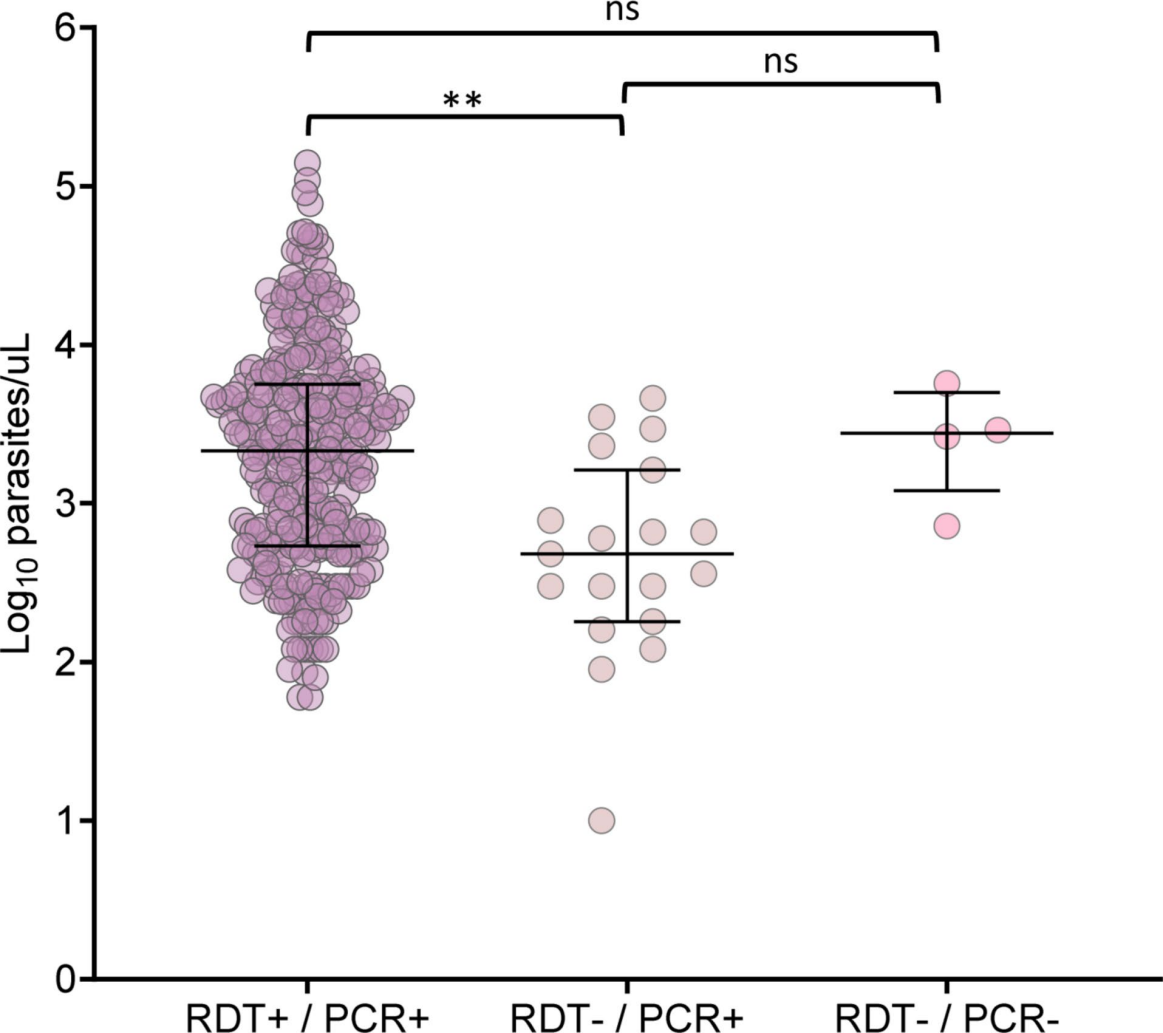
Association between the results of rapid diagnostic tests (RDT), PCR for the detection of *pfhrp2*, and parasitemia. The term PCR refers to the combination of nPCR and qPCR results. ns, not statistically significant (by Dunnett’s post hoc test).

All 365 samples were amplified for the internal control (*pfrnr2e2*) of the reaction in the multiplex qPCR. For *pfhrp2*, only 5 samples did not amplify by qPCR (Fig. 3a). Four samples (1%) were negative for both molecular tests (nPCR and qPCR), including the RDT (Fig. 3b). No samples were positive for the RDT alone. Of the four samples that did not amplify for any diagnostic method, the MAL7P1.228 flanker was present in all isolates, unlike the MAL7P1.230 flanker. For the subsequent analyses, the results from nPCR and qPCR were combined and referred to as PCR. Samples were classified as negative only if they did not amplify in either method. The results obtained by PCR and RDT were combined (RDT+/PCR+; RDT−/PCR+ and RDT−/PCR−) to evaluate the influence of parasitemia on their positivity. There was no significant difference in parasitemia between the positive and negative groups for both tests, ruling out the possibility of this factor influencing the negative results for both tests (Fig. 4). In the RDT−/PCR+ group, parasitemia was significantly lower compared to the other two groups (*P* = 0.001, by Dunnett’s post hoc test).

### Characterization of the sequence pattern of the *pfhrp2* gene

A comprehensive analysis of the genetic diversity of HRP2 was conducted by successfully sequencing 94 samples representing the *P. falciparum* isolates circulating in the studied areas. We included all samples with divergent results between RDT and PCR (RDT−/PCR+), as well as randomly selected positive samples for both tests. Positive samples for only one of the molecular methods (qPCR or nPCR) were also sequenced, confirming the presence of *pfhrp2*. All sequences began with the type 1 repeat (AHHAHHVAD) and ended with the type 12 (AHHAAAHHEAATH). Types 2 (AHHAHHAAD) and 7 (AHHAAD) occurred more frequently per sample (Supplementary Data 3). We identified eight different sequence patterns based on the type and number of repeat units. The most prevalent was the pattern I observed in 39 (42%) isolates, followed by pattern XVII in 26 (28%) and pattern VI in 21 (22%) isolates. Three previously unnamed sequences were identified as patterns XIX, XX, and XXI (occurring in 1 isolate each).

### Confirmation of the deletion of *pfhrp2* gene by ELISA

**Fig. 5:**
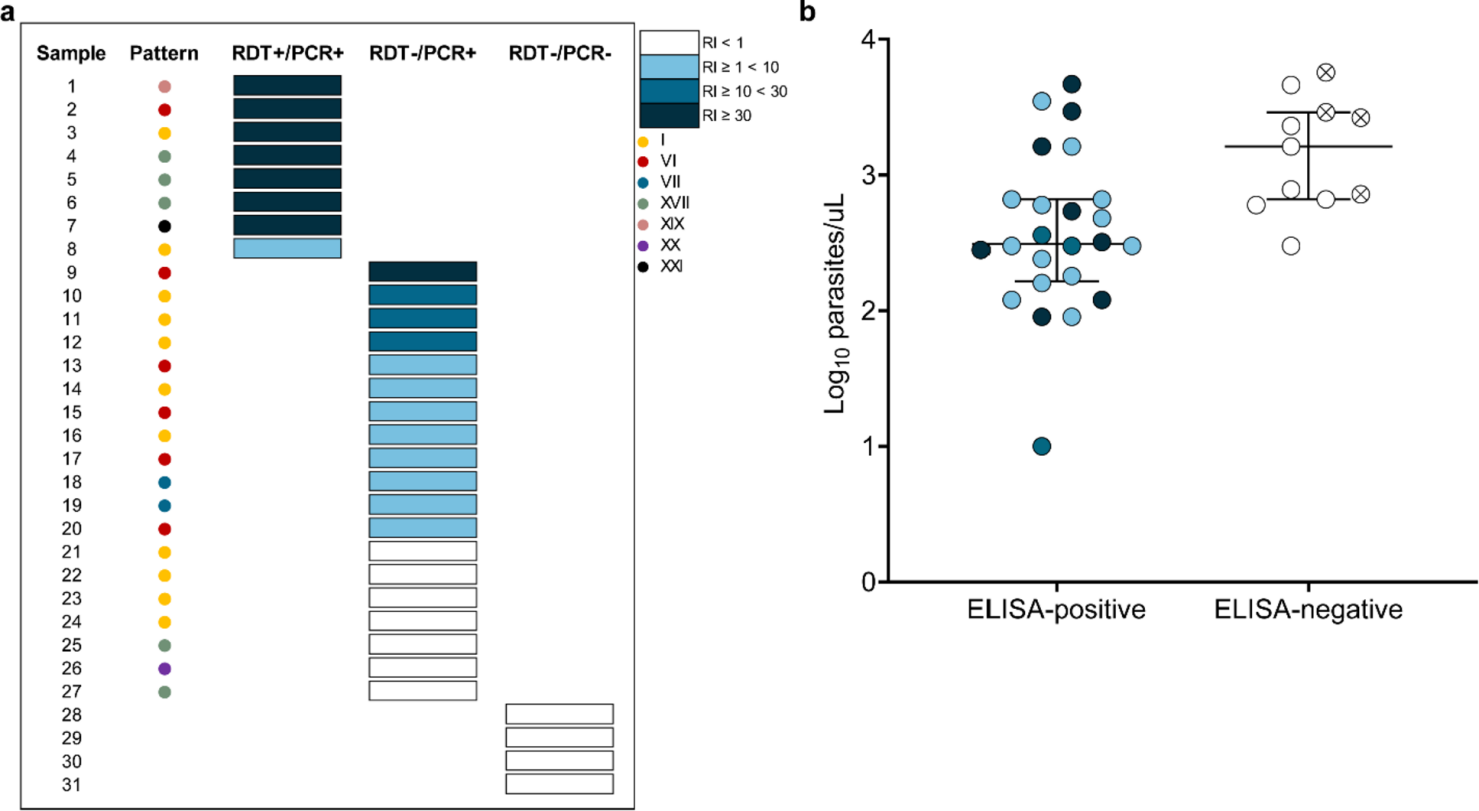
Analysis of HRP2 detection by ELISA, *pfhrp2* genetic diversity, and parasite density by microscopy for 31 samples, with divergent results between RDT and PCR. **(a)** The association between HRP2 detection by ELISA and the results obtained by RDT and PCR. The reactivity indices (RI) values were divided into four ranges, each represented by a different color. The blue color, ranging from darkest to lightest, respectively, represents RI ≥ 30, RI ≥ 10 < 30, RI ≥ 1 < 10, with RI < 1 in white representing negative results. The first column indicates the sequence pattern of the HRP2 protein, as determined by sequencing. PCR refers to the combination of the results obtained from nPCR and qPCR. **(b)** Association between HRP2 detection by ELISA and parasitemia. Samples marked with an X represent those with a *pfhrp2* deletion.

The presence of the HRP2 antigen was evaluated for samples that had been tested negative by PCR and RDT, as well as those that had divergent results. Furthermore, seven positive samples, selected at random, were evaluated using both methods. Among the 31 samples analyzed, 11 were considered negative in ELISA. The samples that were positive for both RDT and PCR presented the highest values of reactivity index (RI) (Fig. 5a). Among the 19 RDT−/PCR + divergent samples, 12 (63%) were found to be reactive in the ELISA. Therefore, 7 (35%) of the PCR-positive samples were RDT− and non-reactive in ELISA. It was not possible to establish a clear association between the HRP2 sequence pattern and reactivity in the ELISA (Fig. 5a). In addition, no association was found between blood parasitemia levels and ELISA reactivity (Fig. 5b). In general, ELISA-negative samples showed higher levels of blood parasitemia (median = 1620 parasites/µL, IQR = 660–2900 for ELISA-negative compared to median = 300 parasites/µL, IQR = 160–660 for ELISA-positive).

### Analysis of intra-host genetic diversity

All samples with divergent results between RDT, PCR, and ELISA were evaluated for the presence of sub-populations of the parasite expressing different levels of HRP2 expression. In total, 31 samples (same as the previous section) were successfully genotyped for the IC/3D7 and FC27 merozoite surface protein 2 (*msp2*) families. Of these, 23 (74%) were defined as monoclonal infections. The IC/3D7 family was the only one that was present in all isolates, while the FC27 family was observed in only 2 (6%) (Supplementary Data 4). Only 1 sample presented polyclonal infection among 5 samples with divergent results (RDT−/PCR+) and with parasitemia > 1000 parasites/µL. For this sample, the presence of genetically distinct parasites could explain the divergence between the tests. In the case of the remaining samples, the lack of protein detection by RDT may be attributed to the low parasitemia levels observed. Also, none of the non-reactive samples in ELISA (RDT−/PCR+) showed polyclonal infection among those with high parasitemia (> 1000 parasites/µL).

## Discussion

Rapid diagnostic tests represent a crucial tool for accurate and timely malaria diagnosis, particularly in remote areas with poor infrastructure, such as mining sites and indigenous communities in the Brazilian Amazon region. One of the major concerns regarding the use of RDT is the presence of parasites that lack the *pfhrp2/3* genes. The deletion of these genes can lead to a reduction in the efficacy of RDT based on HRP2, potentially resulting in false-negative outcomes. In South America, high prevalences of dual *pfhrp2*/3-deleted parasites have been reported in Peru and Brazil^9–12^. The WHO recommends the continued surveillance of areas where the *pfhrp2/3* deletions have been reported and the implementation of non-HRP2-based diagnostics if the prevalence of *pfhrp2* deletion causing false-negative RDT results exceeds the threshold of 5%. In this study, we conducted a comprehensive survey of *pfhrp2/3* deletions in Roraima State, in the northern region of Brazil. This area is part of the Guiana Shield, which is currently one of the main hotspots for malaria transmission in the Americas and has significantly contributed to the regional migratory movement on the Brazil-Venezuela-Guyana tri-border.

We performed molecular (nPCR and qPCR) and immunological (RDT and ELISA) methods for detecting HRP2 and confirming *pfhrp2/3* deletions. In general, greater *pfhrp2* positivity was observed in molecular methods compared with protocols that evaluate the presence of HRP2. The combined results of PCR, indicate a very low prevalence (1%) of *pfhrp2* deletion in *P. falciparum* isolates circulating in the studied areas. The absence of amplification for one or both flanking genes in parasites lacking *pfhrp2* confirmed the occurrence of a genome deletion extending beyond the *pfhrp2* gene. The absence of HRP2 was confirmed by the immunological methods. Here, we identified a higher prevalence of *pfhrp3*-deleted parasites, which is in accordance with the findings of other studies that have made similar observations^13–16^. Although the overall prevalence of *pfhrp2*-deleted parasites is low, these isolates also lacked the *pfhrp3* gene, thereby eliminating the possibility of cross-reactivity between anti-HRP2 antibody and HRP3, and, consequently, the possibility of detection by RDT.

The prevalence of *pfhrp2* deletion observed here differed significantly from that reported recently by Costa et al. These authors reported a prevalence of ≅ 15% for samples also collected in Roraima State^10^. It is important to note that the samples analyzed by Costa et al. were collected more recently (2018–2020) than those analyzed in the present study (2016–2018). Most of the subjects in the present study were infected in gold mining areas in Venezuela. The period of sample collection coincides with the intense migratory movements from Venezuela to Brazil between 2015 and 2017, which followed an economic crisis that affected the neighboring country^17,18^. In turn, this scenario has contributed to a significant increase in illegal mining activity in the Amazon.

A detailed analysis of the HRP2 sequences revealed a significant shift in their overall profile. Patterns XVII and VI were found in approximately 50% of the isolates analyzed, which had not been previously identified in this area. Pattern XVII has been reported only in French Guiana, while pattern VI has been observed in the northeast Brazilian Amazon (Amapá state) and French Guiana^10,19^. On the other hand, the pattern I was the most prevalent in this area, representing approximately 40% of the total observations. This pattern has also been previously described in areas of the Brazilian and French Guiana border regions^10^. No clear association could be identified between HRP2 sequence patterns and the performance of RDT or ELISA. For instance, both patterns I and XVII were found to be associated with both negative and positive results in RDT or ELISA. It is beyond the scope of the current study to ascertain whether HRP2 sequence variability affects the performance of the RDT.

In the present study, the performance of the RDT was found to be limited by its inability to detect the parasite at low densities. Among the 19 samples that presented divergent results between RDT and PCR (RDT−/PCR+), 14 presented low parasitemia, with levels ranging from 10 to 780 parasites/µL. An investigation was carried out for the remaining samples with parasitemia above 1000 parasites/µL to determine if the presence of parasite populations with and without *pfhrp2* deletion in the same infection could account for the observed differences. As a result, if the predominant isolate has undergone a *pfhrp2* deletion or if only very low levels of HRP2 are present, an RDT may fail to detect a high parasitemia infection. In this case, only one RDT−/PCR + sample was identified as a polyclonal infection. Furthermore, samples that tested negative by ELISA and positive by PCR were identified as monoinfection. A limitation of this study is that the complexity of infection was assessed by genotyping only *msp2*, which reduced the ability to capture the full genetic variability. Accordingly, we found low genetic diversity, with most infections exhibiting a monoclonal pattern. For now, we cannot rule out the possibility of low expression of HRP2 by the isolates present in these infections with high parasitemia and non-reactive to RDT or ELISA^20^.

In conclusion, our findings indicate a markedly low prevalence (1%) of *pfhrp2*-deleted parasites in the Brazilian Amazon region in the north-central Guiana Shield. Since the prevalence of false-negative RDT results caused by *pfhrp2* deletions is below the WHO threshold of 5%, there is no recommendation to replace the RDT tests used in the study area. Therefore, the presence of 6% of false-negative HRP2-RDT in our study is more likely to be explained by a lower parasite density that can still be detected by the more sensitive PCR method. Low prevalences of *pfhrp2*-deleted parasites have been reported in other areas in the northeast of Brazil, a completely different epidemiological scenario from that reported on the border of Peru^9–12^. However, it is imperative that further surveys be conducted to enable timely detection of *pfhrp2* deletion in this area, which is characterized by a high number of cross-border malaria cases, and implement corrective measures if indicated.

## Methodology

The samples used in this study are part of a broad cross-sectional study that sought to describe the epidemiological pattern of malaria through spatial analysis of factors linked to the disease. The ethical and methodological aspects of this study were approved by the Ethics Committee of Research Involving Human Subjects of the Institute René Rachou/Fiocruz (CAAE: 70755617.8.0000.5091). Prior to their participation in the study, subjects were informed regarding the objectives and procedures of the study, and their voluntary participation was requested and confirmed through written formal consent. The study was performed in accordance with all relative guidelines and regulations.

### Study design and data collection

This was an observational, cross-sectional study performed in Roraima between March 2016 and September 2018. In 2016, the number of malaria cases in Roraima State was 5,729, including 466 cases of *P. falciparum*, while in 2017 and 2018, this number increased to 11,203 (202 *P. falciparum* cases) and 18,371 (687 *P. falciparum* cases), respectively^21^. Blood samples were collected from individuals who sought public healthcare services in the cities of Boa Vista, Rorainópolis, and Pacaraima in the state of Roraima, Brazil. The subjects were symptomatic and had sought treatment from the local public health services. The presence of *Plasmodium* spp. infection was confirmed by microscopy, based on Giemsa-stained thick blood smears, which were evaluated by well-trained microscopists according to the guidelines for malaria diagnosis of the Brazilian Ministry of Health. Following the confirmation of infection, patients positive for *P. falciparum* were invited to participate in the research (Supplementary Data 1). The parasite density was determined as the number of asexual parasites observed per 200 leukocytes on a thick smear, with an estimated leukocyte count of 6,000 per µL. Subsequently, frozen blood samples were subjected to the SD-Bioline™ Malaria Ag Pf/Pf/Pv (Abbott, Inc.; Korea) test, which employs the proteins HRP2 and LDH as targets for the detection of *P. falciparum*. All samples were tested for malaria infection using a PET-PCR assay, as previously described by Lucchi et al.^22^. Firstly, infection by the *Plasmodium* genus was identified by the 18 S ribosomal target, and then infection by *P. falciparum* was identified by amplification of the Pfr364 target from the primers described in Demas et al.^23^.

### Nested PCR (nPCR) assays for amplification of the *pfhrp2*/*3* and their flanking genes

DNA was extracted from 200 µL whole blood samples using the QIAamp DNA Mini Kit (QIAGEN, Minneapolis, MN, USA) according to the manufacturer’s instructions. The DNA was eluted in 50 µL of elution buffer to perform molecular assays. To evaluate the presence of *pfhrp2* (PF3D7_0831800) and *pfhrp3* (PF3D7_1372200), nPCR was performed using the primers and conditions described in Abdallah et al.^13^ on the Veriti^®^ thermal cycler (Applied Biosystems). Samples that did not amplify for *pfhrp2* and *pfhrp3* were subjected to re-amplification to confirm the gene deletion. The flanking genes upstream and downstream to *pfhrp2* (MAL7P1.230 and MAL7P1.228) and *pfhrp3* (MAL13P1.475 and MAL13P1.485) were amplified for all negative samples, also using the conditions previously described in Abdallah et al.^13^. The amplified fragments were visualized by electrophoresis in 2% agarose gels in 1x TAE buffer containing 5 µg/mL of ethidium bromide (Invitrogen) on a horizontal system (Bio-Rad) at 100 V. The gels were examined under UV transillumination, and the images were captured using a digital system (UVP—Bio-Doc System). The laboratory strain 3D7 was used as a positive control for the PCR analyses of *pfhrp2*, *pfhrp3*, and their respective flanking genes.

### *pfhrp2* multiplex quantitative PCR (qPCR)

A second protocol for *pfhrp2* amplification was performed using multiplex qPCR with the primers and probes described by Abdallah et al.^13^ and Schindler et al.^24^, respectively. The single-copy gene *pfrnr2e2* (PF3D7_1015800) was employed as an internal control for the reaction. The reactions were prepared with 1X GoTaq Probe master mix (Promega), 300 nM of each primer and 150 nM of each probe (FAM-labeled probe for *pfhrp2* and VIC-labeled for *pfrnr2e2*), 2 µL sample DNA (~ 10 ng), and RNAse-free water to a final volume of 10 µL. The thermal cycling process was conducted under the following conditions: 50 °C for 2 min, 95 °C for 10 min, followed by 45 cycles of 95 °C for 15 s, 56 °C for 30 s, and 60 °C for 30 s on the QuantStudio™ 12 K Flex Real-Time PCR System. A second qPCR was performed on samples that failed to amplify for *pfhrp2* to confirm the gene deletion.

### *pfhrp2* sequencing and analysis

A conventional PCR was performed using the conditions previously described for the sequencing of the histidine and alanine repetitive regions of *pfhrp2*^25^. The PCR products were purified using the QIAquick PCR purification kit (QIAGEN, Chatsworth, CA, USA) and subsequently analyzed in the ABI 3730xL DNA Analyzer system (Thermo Fisher Scientific, Waltham, MA, USA). The nucleotide sequences were aligned and translated into amino acids using the Mega X software^26^, and the amino acid repeat types were identified using the numerical code described by Baker et al.^25,27^.

### *msp2* genotyping and genetic variation

The central region of *msp2* (IC and FC27) was genotyped by nPCR using primers and reaction conditions previously described in Snounou et al.^28^ and Liljander et al.^29^ to assess infection complexity (presence of multiple genetically distinct parasites). The reactions were subsequently analyzed by capillary electrophoresis using the ABI 3730xL DNA Analyzer system, with the products analyzed using GeneMapper™ software version 4.1 (Thermo Fisher Scientific, Waltham, MA, USA). For the analysis, the minimum peak height was set at 200 arbitrary fluorescence units (rFU), and a threshold of one-third the height of the predominant peak (the peak with the greatest fluorescence) was established to exclude any potential artifacts.

### HRP2 immunological assays

The presence of HRP2 antigen in the blood was assessed by enzyme-linked immunosorbent assay (ELISA) using a commercially available kit (Quantimal Celisa PfHRP2 Assay kit, Cellabs, Cat number: KM8). The positivity cut-off value was determined by subtracting the average optical density (OD) at (450 nM) analyzed on Varioskan LUX Multimode Microplate Reader (Thermo Fisher Scientific, Waltham, MA, USA) of the negative controls from the blank (containing only the reagents and RPMI) and adding one unit. The reactivity index (RI) value was calculated from the ratio between the mean ODs of the samples in duplicates and the cut-off value. To correct variations between experiments, a normalization factor was calculated as the ratio between the OD of the positive control in each experiment and the mean of the readings. Finally, the RI was divided by the corrected value for the day of the experiment, obtaining the normalized index.

## Electronic supplementary material

Below is the link to the electronic supplementary material.

Supplementary Material 1

## Data Availability

The datasets presented in this study can be found in online repositories. The names of the repository/repositories and accession number(s) can be found below: GenBank PP976113, PP976114, PP976115, PP976116, PP976117, PP976118, PP976119, PP976120.
