# Supplementary material for: Very low prevalence of Plasmodium falciparum histidine-rich protein 2 (pfhrp2) gene deletion in the Brazil, Venezuela, and Guyana tri-border
Source: Sci Rep. 2025 Jan 3;15:669. doi: 10.1038/s41598-024-83727-3 (PMC11699284; doi:10.1038/s41598-024-83727-3)
Supplement: Supplementary file 1 — Supplementary Material 1 [file 41598_2024_83727_MOESM1_ESM.pdf]

SUPPLEMENTARY MATERIAL

Supplement to: **Malaria Rapid Diagnostic Tests: very low prevalence of *Plasmodium falciparum* *pfhrp2* gene deletion in the Brazil, Venezuela, and Guyana tri-border**

Maria Eduarda Pereira Mascarenhas, Jaime Louzada, Renato Amorim Rosa, Gabriela Maíra Pereira de Assis, Flora Satiko Kano, Joseli de Oliveira Ferreira, Tais Nobrega de Sousa

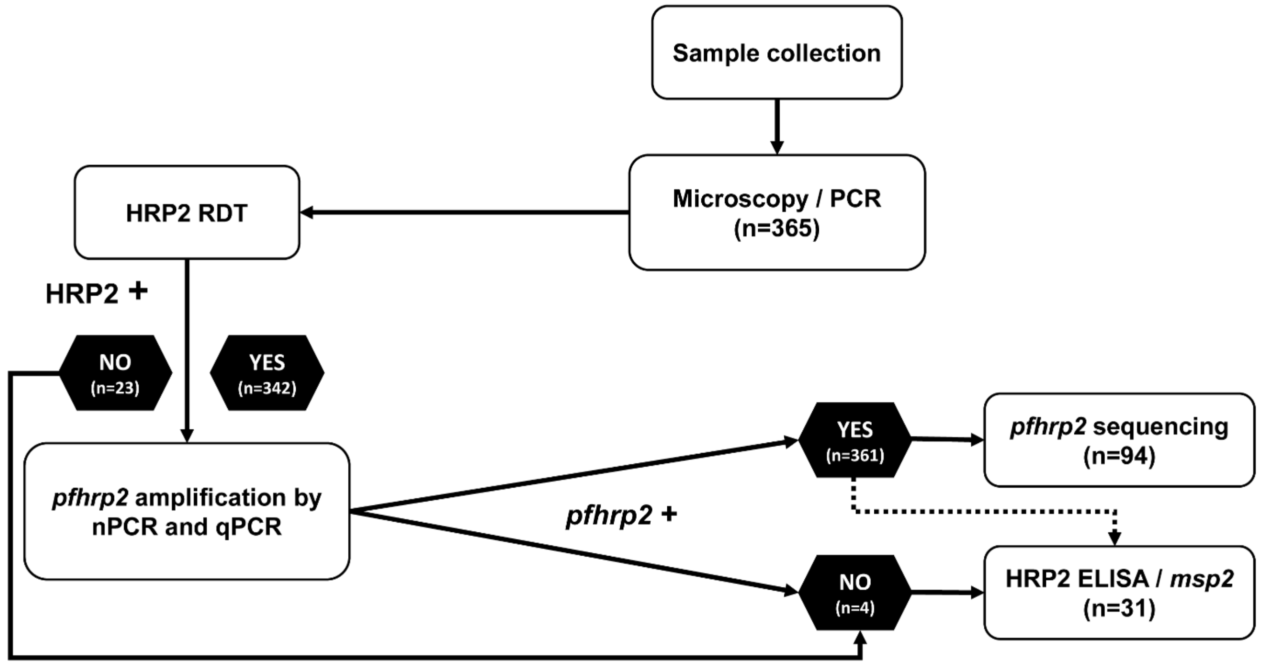

Supplementary data 1. Methodology flowchart. Flowchart covering the different methodologies used to carry out the study with number of samples submitted to each one.

Supplementary data 2. Amplification profile of *pfhrp2/3* and their flanker genes.

Mal7P1.230	*pfhrp2*	Mal7P1.228	N (%)	Mal13P1.475	*pfhrp3*	Mal13P1.485	N (%)
Present	Absent	Present	2(14)	Present	Absent	Present	3(3)
Present	Absent	Absent	5(36)	Present	Absent	Absent	42(42)
Absent	Absent	Present	5(36)	Absent	Absent	Present	3(3)
Absent	Absent	Absent	2(14)	Absent	Absent	Absent	53(52)

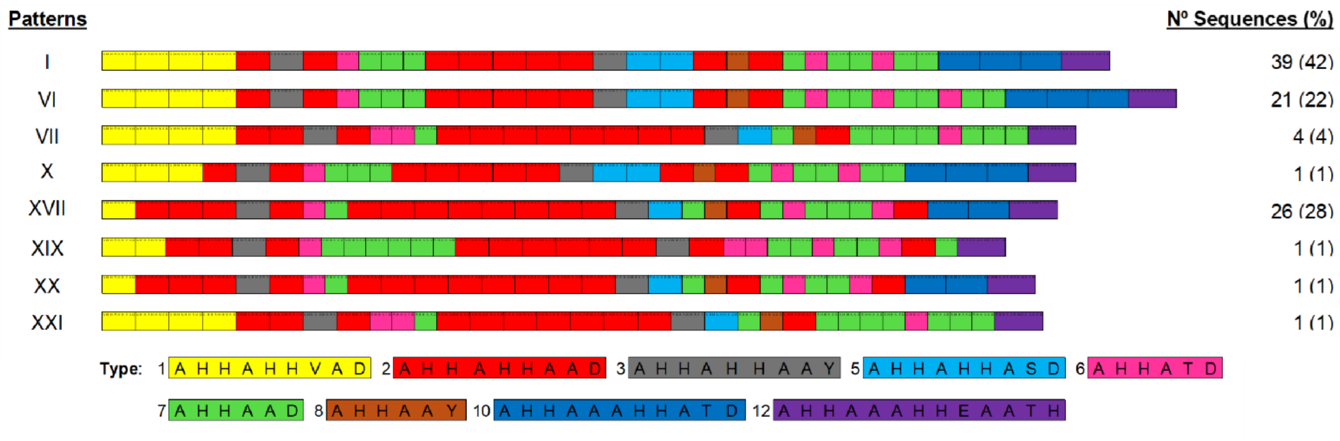

Supplementary data 3. PfHRP2 sequence patterns. Amino acid repeat types were identified as previously described (Baker et al., 2005; Baker et al., 2010). Sequence patterns I to XVII were previously named (Costa et al., 2021), while patterns XIX, XX and XXI were named in the present study.

Supplementary data 4. Intra-host diversity based on *msp2* genotyping. Association between the alleles found for *msp2* families IC and FC27, and the results obtained by molecular and immunological methods for detection of HRP2.

**Sample**	***msp2* IC/3D7**	***msp2* FC27**	**Test results**	**Parasitemia (p/uL)**
1	532	*	RDT+/PCR+/ELISA+	90
6	532	*	RDT+/PCR+/ELISA+	120
8	532	*	RDT+/PCR+/ELISA+	240
5	532	*	RDT+/PCR+/ELISA+	280
7	532	*	RDT+/PCR+/ELISA+	320
2	532	*	RDT+/PCR+/ELISA+	540
3	532	*	RDT+/PCR+/ELISA+	1,620
4	532	*	RDT+/PCR+/ELISA+	4,680
12	532	294	RDT-/PCR+/ELISA+	10
13	440; 532	*	RDT-/PCR+/ELISA+	90
19	532	*	RDT-/PCR+/ELISA+	120
18	532	*	RDT-/PCR+/ELISA+	160
14	440; 532	*	RDT-/PCR+/ELISA+	180
10	440; 532	*	RDT-/PCR+/ELISA+	300
16	440; 532	*	RDT-/PCR+/ELISA+	300
11	532	*	RDT-/PCR+/ELISA+	360
17	532	*	RDT-/PCR+/ELISA+	480
15	440; 532	*	RDT-/PCR+/ELISA+	660
9	440; 532	*	RDT-/PCR+/ELISA+	2,940
20	532	*	RDT-/PCR+/ELISA+	3,500

**Sample**	***msp2* IC/3D7**	***msp2* FC27**	**Test results**	**Parasitemia (p/uL)**
24	532	294	RDT-/PCR+/ELISA-	300
25	532	*	RDT-/PCR+/ELISA-	600
26	440; 532	*	RDT-/PCR+/ELISA-	660
22	644	*	RDT-/PCR+/ELISA-	780
27	532	*	RDT-/PCR+/ELISA-	1,620
21	532	*	RDT-/PCR+/ELISA-	2,300
23	644	*	RDT-/PCR+/ELISA-	4,600
30	519	*	RDT-/PCR-/ELISA-	720
29	644	*	RDT-/PCR-/ELISA-	2,640
31	532	*	RDT-/PCR-/ELISA-	2,900
28	519; 532	*	RDT-/PCR-/ELISA-	5,702
